# Evaluation of protein extraction methodologies on bacterial proteomic profiling: a comparative analysis

**DOI:** 10.3389/fmicb.2025.1586662

**Published:** 2025-07-17

**Authors:** Hongning Jiang, Aiyun Han, Yangdong Zhang, Yanxin Li, Chao Jiang, Qijing Du, Rongbo Fan, Yongxin Yang, Rongwei Han

**Affiliations:** ^1^College of Food Science and Engineering, Qingdao Agricultural University, Qingdao, China; ^2^Shijiazhuang Food Engineering Technology Innovation Center, Shijiazhuang University, Shijiazhuang, Hebei, China; ^3^Key Laboratory of Quality & Safety Control for Milk and Dairy Products of Ministry of Agriculture and Rural Affairs, Institute of Animal Sciences, Chinese Academy of Agricultural Sciences, Beijing, China; ^4^Shandong Sanyuan Dairy Co., Ltd., Weifang, Shandong, China

**Keywords:** microbial proteomics, protein extraction optimization, data-dependent acquisition, data-independent acquisition, proteomic methodology

## Abstract

Bacterial proteomics is a pivotal tool for elucidating microbial physiology and pathogenicity. The efficiency and reliability of proteomic analyses are highly dependent on the protein extraction methodology, which directly influences the detectable proteome. In this study, we systematically compared four protein extraction protocols—SDT lysis buffer with boiling (SDT-B), SDT lysis buffer with ultrasonication (SDT-U/S), a combination of boiling and ultrasonication (SDT-B-U/S), and SDT lysis buffer with liquid nitrogen grinding followed by ultrasonication (SDT-LNG-U/S)—to evaluate their effects on peptide and protein identification, distribution, and reproducibility in *Escherichia coli* and *Staphylococcus aureus*. Both data-dependent acquisition (DDA) and data-independent acquisition (DIA) strategies were employed for comprehensive proteomic profiling. DDA analysis identified 23,912 unique peptides corresponding to 2,141 proteins in *E. coli* and 13,150 unique peptides corresponding to 1,511 proteins in *S. aureus*. DIA analysis yielded slightly fewer peptides (21,027 for *E. coli* and 7,707 for *S. aureus*) but demonstrated superior reproducibility. Among the tested protocols, SDT-B-U/S outperformed the others, identifying 16,560 peptides for *E. coli* and 10,575 peptides for *S. aureus* in DDA mode. It also exhibited the highest technical replicate correlation in DIA analysis (*R*^2^ = 0.92). This method enhanced the extraction of proteins within key molecular weight ranges (20–30 kDa for *E. coli*; 10–40 kDa for *S. aureus*) and was particularly effective for recovering membrane proteins (e.g., OmpC). Additionally, ultrasonication-based protocols outperformed the liquid nitrogen grinding approach in extracting the *S. aureus* proteome. These findings underscore the significant impact of protein extraction methods on bacterial proteomics. The SDT-B-U/S protocol—thermal denaturation followed by ultrasonication—proved most effective, enhancing protein recovery and reproducibility across both Gram-negative and Gram-positive bacteria. This work offers key guidance for optimizing microbial proteomic workflows.

## 1 Introduction

Proteomic technologies have advanced considerably in recent years, enabling a wide range of analytical approaches and applications. However, the effectiveness of metaproteomic analyses is highly contingent upon the protein extraction methods employed, particularly with respect to protein yield and the accurate representation of bacterial species within complex microbial communities. Protein sample preparation is a critical initial step in proteomic workflows, as it directly affects the accuracy and depth of protein identification and quantification (Andersen et al., 2021). The inherent complexity of bacterial proteomes—characterized by wide-ranging protein abundances and diverse physicochemical properties—further underscores the need for optimized extraction strategies (Dupré et al., 2020).

Currently, a variety of extraction techniques are utilized for both Gram-negative and Gram-positive bacteria, including enzymatic, chemical, thermal, and mechanical disruption methods such as magnetic bead homogenization and ultrasonication (Kielkopf et al., 2021; Palma Medina et al., 2019; Zhang et al., 2016; Tian et al., 2022). Each approach has inherent limitations: for example, ultrasonication generates heat that can denature thermolabile proteins (Yusaf, 2015), while glass-bead milling, though efficient in protein yield, produces excessive cell debris that may interfere with downstream analysis (Haberl Meglič et al., 2020). Lysozyme, a commonly used enzyme, is less effective against Gram-negative bacteria unless combined with outer membrane permeabilizers such as polymyxin B or chlorhexidine, and typically requires extended incubation times and specific concentrations (Ghose and Euler, 2020). Sodium dodecyl sulfate (SDS), a widely used anionic detergent, plays a pivotal role in electrophoresis and cell lysis for proteomics (Arakawa et al., 2024). Numerous studies have employed SDS-based buffers for bacterial protein extraction. For instance, Crowell et al. (2015) utilized SDS lysis combined with boiling to extract total proteins from *E. coli*. Similarly, Song et al. (2023) incorporated SDS and ultrasonication, successfully identifying 1,949 to 2,118 proteins from 96 wild-type *Cronobacter* strains. Xu et al. (Xu et al., 2021) identified a lysin protein PlyEc2 by sonicating *E.coli* in an ice bath. Kiser et al. (2007) utilized liquid nitrogen grinding to extract proteins from *E. coli* in a study of GroEL structure.

In contrast to Gram-negative bacteria, Gram-positive bacteria possess a thicker peptidoglycan layer in their cell walls, which presents additional challenges for efficient protein extraction (Ratna et al., 2025). Ultrasonication is generally less efficient for Gram-positive bacteria, often yielding only one-third the protein compared to *E. coli* under similar conditions (Yusaf, 2015). Bead beating also requires longer durations and higher energy input to achieve comparable results (Lee et al., 2022). Although lysozyme is more effective against Gram-positive species, it is expensive, can degrade target proteins, and shows variable efficacy across strains (Bi et al., 2020). Therefore, combination strategies are often necessary to enhance lysis efficiency. One commonly adopted method is the use of SDS-containing lysis buffer prior to sonication. Suo et al. (2018) used this strategy to investigate proteins involved in the resuscitation of frozen S.aureus cells, while Du et al. (2020) applied SDS-based lysis and sonication to analyze methicillin-resistant *S. aureus* (MRSA), successfully identifying 1,499 proteins—nearly matching the transcriptomic dataset.

As mentioned above, SDS is crucial for sample preparation, yet variations exist in protein identification across different studies due to the extraction protocols used. Notably, a comprehensive review of the effects of various protein extraction techniques on bacterial proteomic analysis has yet to be conducted. Consequently, it is essential to assess optimized methods for bacterial protein sample preparation in future research to mitigate the influence of extraction techniques on proteomic studies.

Previous studies primarily focused on single bacterial species or lacked systematic comparisons between Gram-positive and Gram-negative bacteria. This gap limits the development of universally applicable extraction protocols for diverse microbiomes. In this study, researchers systematically evaluated four distinct bacterial protein extraction methods using both data-dependent acquisition (DDA) and data-independent acquisition (DIA) proteomic analyses in *S.aureus* and *E.coli*, which were selected as representative Gram-positive and Gram-negative bacteria, respectively. This is the first study to comprehensively evaluate protein extraction efficacy across both Gram categories using dual proteomic acquisition modes (DDA/DIA), addressing a critical methodological gap in microbial proteomics.

## 2 Materials and methods

### 2.1 Protein preparation

#### 2.1.1 Bacterial strains and pre-treatment

*E.coli* (ATCC 25922) and *S. aureus* (ATCC 25923) were selected as model organisms for this study. The bacteria were cultured in Luria-Bertani (LB) broth (Hopebio, Shandong, China) and tryptic soybean soup (TSB) (Hopebio, Shandong, China), respectively. Cultures were grown to mid-log phase in 200 mL Erlenmeyer flasks with shaking at 225 rpm and 37°C, as determined by their growth curves. Bacterial cells were harvested by centrifugation at 9,000 × g for 10 min at 4°C, washed three times with phosphate-buffered saline (PBS) to remove residual medium, and stored at 4°C until further use. Three technical replicates were performed for each protein extraction method to ensure reproducibility.

##### 2.1.1.1 SDT lysis buffer coupled with boiled

The SDT lysis buffer, composed of 4% (w/v) SDS, 100 mM dithiothreitol (DTT), and 100 mM Tris-HCl (pH 7.6), was prepared as described by Makkar et al. (1982) with minor modifications. Bacterial cells were resuspended in 5 mL of SDT lysis buffer, vortexed thoroughly, and incubated in a 98°C water bath for 10 min to ensure complete cell lysis and protein release. Cellular debris was removed by centrifugation at 10,000 × g for 10 min at 4°C, and the supernatant was collected for protein precipitation.

##### 2.1.1.2 SDT lysis buffer coupled with ultrasonication

This method was adapted from Hansen et al. (2014) with slight modifications. Bacterial cells were resuspended in SDT lysis buffer, vortexed, and subjected to ultrasonication on ice using an ultrasonic cell disintegrator (ATPIO XO-1000D, China) at 70% amplitude for a total of 5 min (5 seconds on, 8 seconds off per cycle). The lysate was centrifuged at 10,000 × g for 10 min at 4°C, and the supernatant was collected for protein precipitation.

##### 2.1.1.3. SDT lysis buffer combined with boiling and ultrasound treatment

Adapted from McNulty et al. (2013), bacterial cells were resuspended in 5 mL of SDT lysis buffer, mixed thoroughly, and incubated in a 98°C water bath for 10 min. After cooling, the lysate was sonicated on ice under the same conditions as described above. Cellular debris was removed by centrifugation at 10,000 × g for 10 min at 4°C, and the supernatant was collected for protein precipitation.

##### 2.1.1.4 SDT lysis buffer combined with liquid nitrogen grinding for ultrasonic treatment

This method was adapted from Fernández-Acero et al. (2006) with minor modifications. Bacterial cells were transferred to a chilled sterile mortar, ground under liquid nitrogen, and resuspended in SDT lysis buffer. The suspension was sonicated on ice under the same conditions as described above. Cellular debris was removed by centrifugation at 10,000 × g for 10 min at 4°C, and the supernatant was collected for protein precipitation.

For all methods, proteins were precipitated by adding four volumes of pre-cooled acetone to the lysates and incubating overnight at −20°C. The mixtures were centrifuged at 10,000 × g for 10 min at 4°C, and the protein pellets were washed twice with ice-cold acetone. The pellets were resuspended in 100 mM Tris-HCl for protein quantification using a BCA protein assay kit (Thermo Fisher Scientific, IL, USA).

### 2.2 SDS-PAGE analysis

Polyacrylamide gels were prepared using 12% separating gels and 5% concentrating gels, and 20 μg of protein samples were mixed with loading buffer, then heated in a water bath at 95°C for 10 min. After the samples were cooled, the samples and protein markers of 14.4 kDa−97.4 kDa (Beijing Sunshine Bio, China) were added for electrophoresis. Electrophoresis was run at 80v for 20 min, then adjusted to 120 V for 60 min. The gels were stained with Coomassie brilliant blue G-250 solution and then decolorized using distilled water for gel imaging.

### 2.3 Trypsin digestion, desalting

The enzymatic hydrolysis of the sample was done according to the method of Yang et al. (2018, 2025). Briefly, thirty micrograms of bacterial protein samples were mixed with 100 mM Tris-HCl, then DTT was added to a final concentration of 100 mM, and heated in a 50°C water bath for 30 min. After cooling the samples, 200 μL of UT buffer (8 M urea and 100 mM Tris-HCl, pH 8.5) was added and transferred the content to filter tubes (10-kDa cut-off, Sartorius, Goettingen, Germany) and then centrifuged at 14,000 × g for 25 min. The samples were washed with UT buffer, then 100 μL of 50 mM iodoacetamide (IAA) solution was added and incubated for 45 min at room temperature in the dark. Then, the samples were washed three times with 100 μL of UT buffer and two times with 200 μL of 50 mM ammonium bicarbonate solution. Finally, 100 μL trypsin buffer (1 μg sequencing-grade trypsin in 50 mM NH_4_HCO_3_) was added to the washed samples and incubated at 37°C for 16–18 h. The mixture was transferred to a new tube, centrifuged at 14,000 × g for 15 min, and washed twice with 50 mM NH4HCO3. The eluates were collected and stopped by the addition of formic acid (FA) and then desalted using a C18 column (60108-303, Thermo Fisher Scientific, TN, USA). Samples were freeze-dried in a speed vacuum and stored at −80°C.

### 2.4 DDA and DIA analysis by nano-liquid chromatography-tandem mass spectrometry

The dried tryptic peptides were resuspended in 0.1% FA and subjected to the EASY-nLC 1000 coupled with the Orbitrap Fusion Lumos (Thermo Fisher Scientific, Milford, MA, USA). The peptides were loaded to a C18 trap column (100 μm × 20 mm, 5 μm; Thermo Fisher Scientific) using an autosampler and separated by the C18 analytic column (75 μm × 150 mm, 3 μm; Thermo Fisher Scientific) at a flow rate of 300 nL/min. The column was equilibrated with buffer A (water).

For DDA analysis, the mass spectrometry was performed in the positive ion mode with a parent ion scanning range of 350–1,550 Th and automatically switching between MS and MS/MS acquisition. Mass spectrometry parameters are set as follows: (1) MS: resolution: 60,000; AGC target: 200,000; maximum injection time: 50 ms; exclusion duration: 60 s; isolation width 1.6 Th; normalized collision energies: 27 eV. (2) HCD-MS/MS: resolution: 15,000; AGC target = 10,000; maximum injection time: 35 ms.

For DIA analysis, mass spectrometry was performed in the positive ion mode with a parent ion scanning range of 395–1,205 Th. The parameters of mass spectrometry were set as follows: (1) MS: resolution: 60,000; AGC target: 200,000; maximum injection time: 100 ms; (2) HCD-MS/MS: resolution: 60,000; AGC target: 100,000; collision energy: 30 eV. (3) DIA using an isolation width of 26 Da (containing 1 Da for the window overlap) and 26 overlapping windows were constructed covering the precursor mass range of 300–1,550 Da for DIA acquisition.

### 2.5 Proteomic data analysis

#### 2.5.1 Database search for peptide/protein identification and quantification

The DDA raw files were subjected to the MaxQuant software (version 2.0.3.0) to search the database downloaded from the UniProt database (6,604 entries for *E. coli*; 11,217 entries for *S. aureus*; Download December 2021) (Tyanova et al., 2016). The parameters were set as follows: The digestion mode was set to the Trypsin/P specificity, maximum missed cleavages at 2, fixed carbamidomethyl modification of cysteine, and variable modifications of protein N-terminal acetylation and methionine oxidation. Protein and peptide identification was achieved with a false discovery rate (FDR) of 1% of filtered data. The conditions for the match between runs were set as 0.7 match time window, 0.05 ion mobility, 20 alignment time window, and one alignment ion mobility—label-free quantification (LFQ) of identified proteins based on a razor and unique peptide abundance (Nahnsen et al., 2013). The DIA raw files were searched using MaxQuant software against the spectral library established by DDA and the downloaded database and the parameter settings were the same as those applied in the DDA procedure.

#### 2.5.2 Statistical analysis

At least two identified peptides in bacterial proteins and all three replicates of each study group were selected and imported into Perseus software (www.maxquant.org/perseus/). Principal component analysis (PCA) was carried out on log-transformed LFQ intensities of proteomes with values in all samples to eliminate bias from missing values. Hierarchical clustering and PCA analyses were performed on the quantitative proteins of the study group. One-way ANOVA and statistical analyses of identified peptides and proteins followed by Dunnett's multiple comparisons test were performed using GraphPad Prism version 8.0.0 for Windows (GraphPad Software, San Diego, California USA, www.graphpad.com). Differentially abundant proteins were determined based on fold changes ≥2 and *P*-values < 0.05. The identified bacterial proteins associated with the annotation function were analyzed according to the Uniprot website (www.uniprot.org).

## 3 Results

### 3.1 General characterization of bacterial proteins

In this study, we systematically evaluated four distinct protein extraction methods for *E. coli* and *S. aureus* using SDS-PAGE electrophoresis. The resulting gels exhibited protein bands, with no significant differences observed across the extraction methods (Supplementary Figure S1). The protein samples underwent identification via DDA and DIA proteomic techniques. For *E. coli*, the DDA method identified a total of 23,912 unique peptides corresponding to 2,141 proteins, while the DIA method identified 21,027 unique peptides associated with 1,979 proteins (Supplementary Table S1). Similarly, for *S. aureus*, the DDA approach yielded 13,150 unique peptides linked to 1,511 proteins, whereas the DIA method identified 7,707 unique peptides corresponding to 1,143 proteins (Supplementary Table S2).

### 3.2 Characterization of identified peptides and proteins in *E. coli*

**Figure 1:**
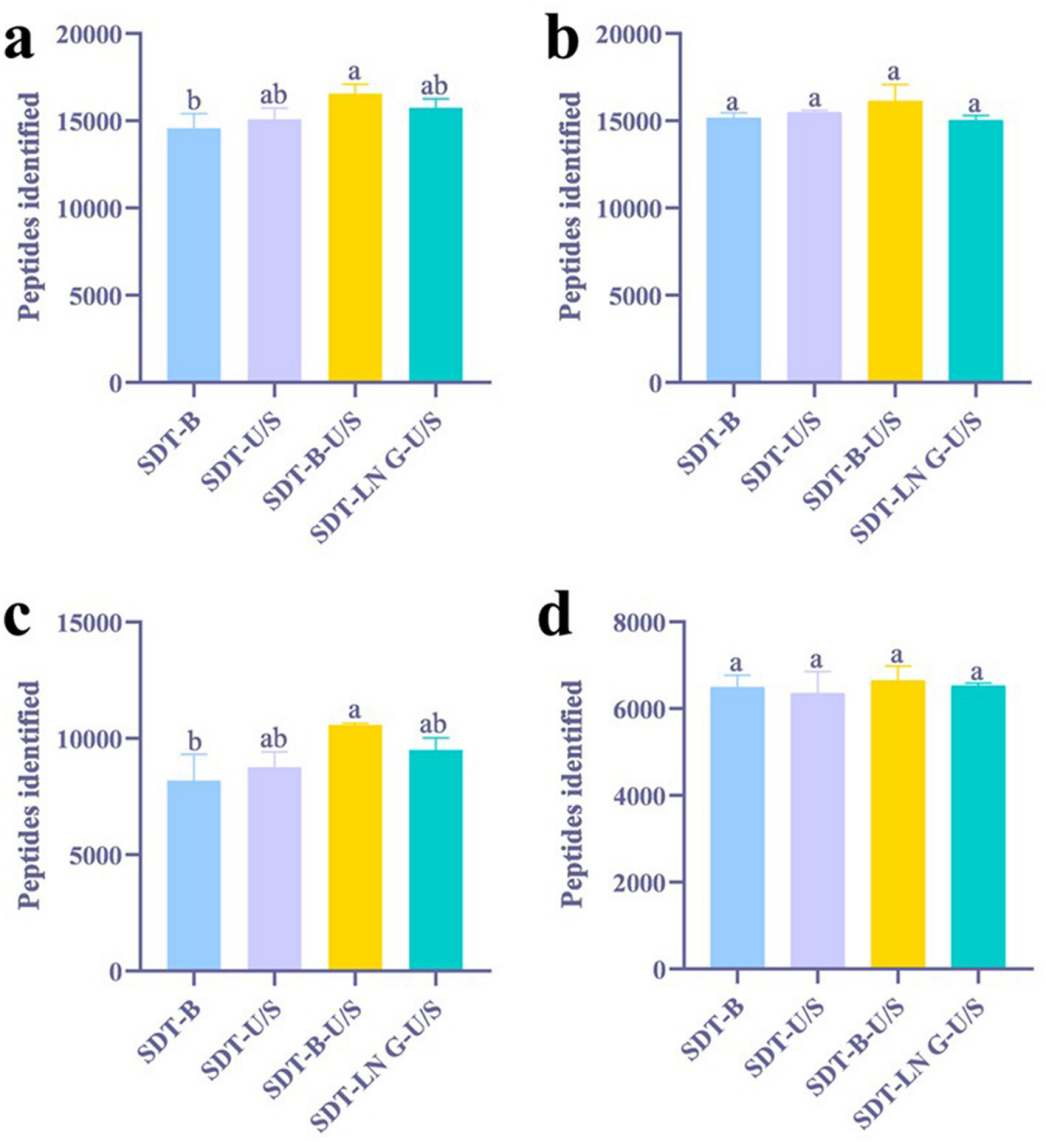
Effects of different treatments on the identification of peptides. **(a)** DDA method for peptide identification of *E. coli*. **(b)** DIA method for peptide identification of *E. coli*. **(c)** DDA method for peptide identification of *S. aureus*. **(d)** DIA method for peptide identification of *S. aureus*. The statistical significance of the four different treatments was assessed using one-way ANOVA and indicated by letters. The groups with different letters had statistically significant differences (*P* < 0.05).

**Figure 2:**
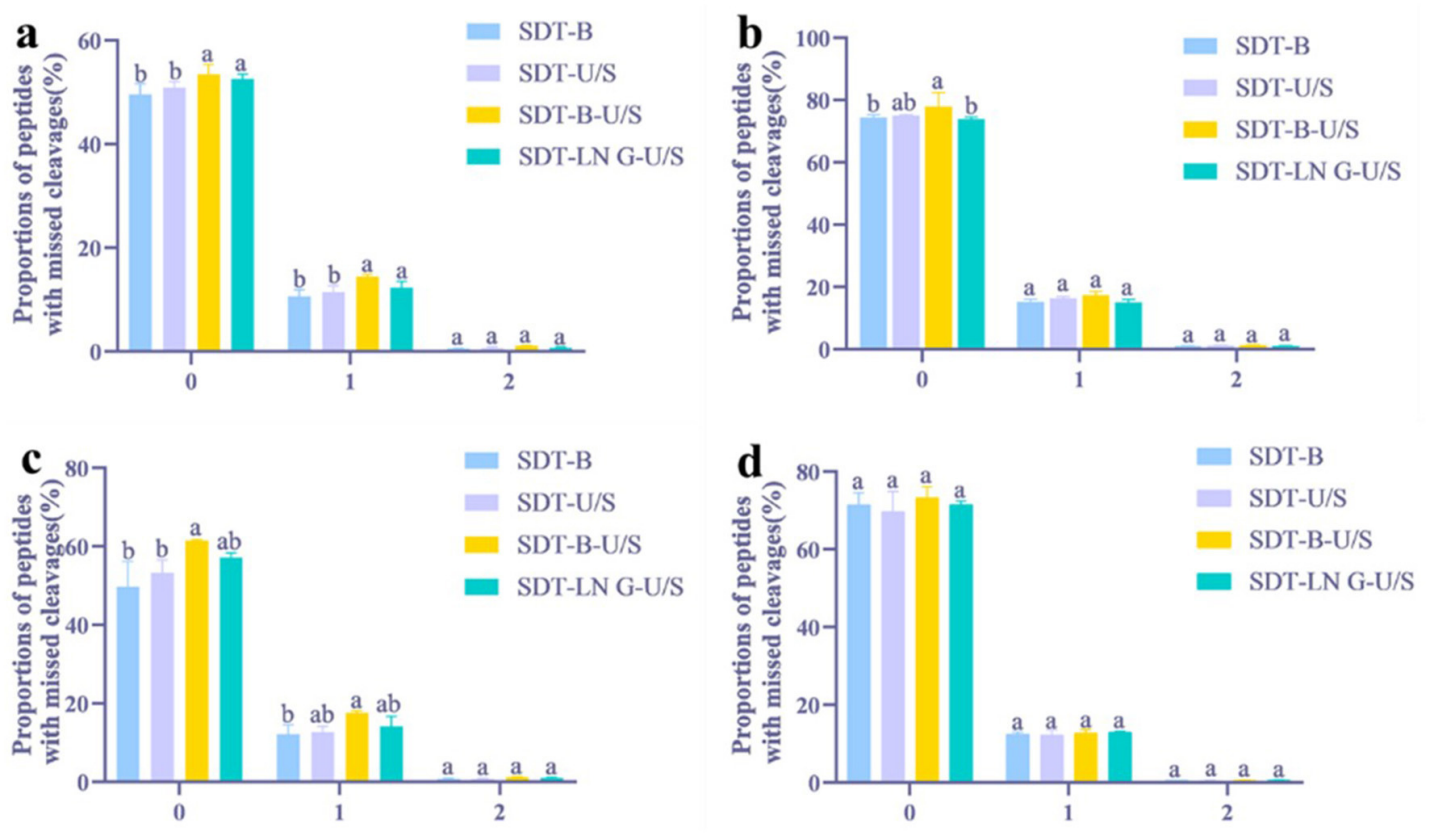
Distribution of peptides with missed tryptic cleavages in four different treatments. **(a)** DDA method for peptide identification of *E. coli*. **(b)** DIA method for peptide identification of *E. coli*. **(c)** DDA method for peptide identification of *S. aureus*. **(d)** DIA method for peptide identification of *S. aureus*. The statistical significance of the four different treatments was assessed using one-way ANOVA and indicated by letters. The groups with different letters had statistically significant differences (*P* < 0.05).

Proteins and peptides identified from *E.coli* using both DDA and DIA methodologies were analyzed using GraphPad Prism software. Among the evaluated extraction methods, the SDT-B-U/S approach yielded the highest number of peptides (16,560) in DDA analysis, representing a notable increase compared to SDT-B (14,572) (Figure 1a). Analysis of tryptic cleavage sites revealed that the occurrence of peptides with 0 and 1 missed cleavage sites was significantly higher in the SDT-B-U/S and SDT-LN G-U/S methods compared to the SDT-B and SDT-U/S methods (Figure 2a).

In the DIA analysis, the number of peptides identified was no significant differences across the four extraction methods (Figure 1b). However, the SDT-B-U/S method demonstrated the highest percentage of peptides missed cleavage sites, while the SDT-B, SDT-U/S, and SDT-LN G-U/S methods showed no significant differences in this regard (Figure 2b).

**Figure 3:**
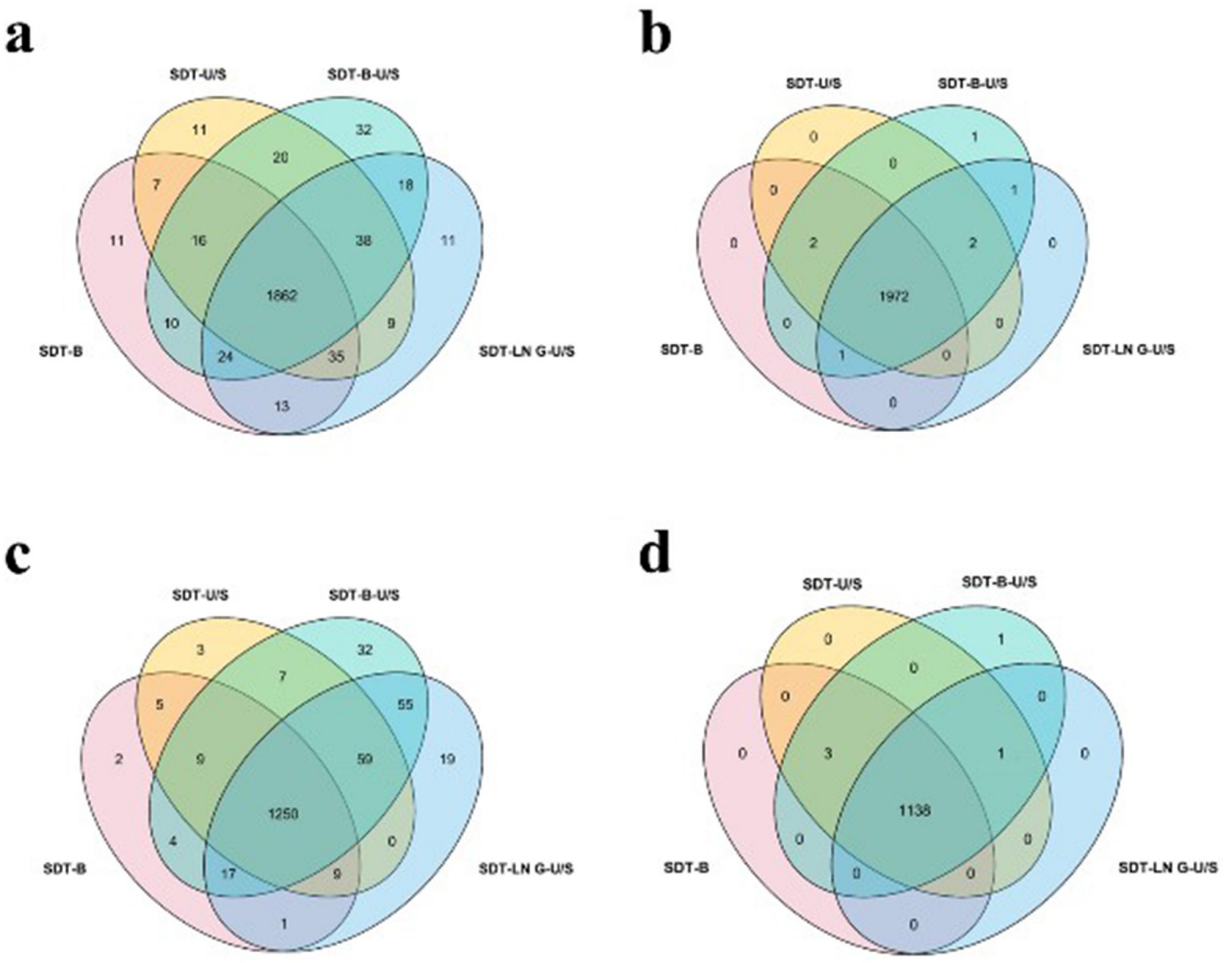
Effect of different treatments on protein quantity identification. **(a)** *E. coli* identified by DDA method. **(b)** *E. coli* was identified by the DIA method. **(c)** *S. aureus* identified by DDA method. **(d)** *S. aureus* identified by the DIA method.

In this study, proteins identified with a minimum of two peptides and three independent runs per group were considered for analysis. The distribution and variability of identified proteins from the four extraction methods, based on the DDA approach, are illustrated in Venn diagrams. A total of 1,862 proteins were identified across the four sample preparation methods, with no significant variation in protein yield observed among them. Among them, 109 proteins were common to three methods, while 65 proteins were unique to a single method (Figure 3a). Regarding the distribution of proteins by molecular weight (MW), the number of proteins within the 20–30 kDa range was significantly higher in samples prepared using the SDT-B-U/S and SDT-LN G-U/S methods compared to the SDT-B and SDT-U/S methods. No statistically significant differences were observed among the methods for proteins in other molecular weight ranges. These findings suggest that proteins of similar molecular weight were successfully extracted from *E.coli* using all four sample preparation techniques. Additionally, the distribution of proteins based on isoelectric point (pI) remained consistent across methods, with the highest number of identified proteins exhibiting pI values of 5.0 and 6.0, regardless of the sample preparation method employed (Table 1).

**Table 1:** The molecular weight, isoelectric point, and subcellular localization of *E. coli* protein are identified from the four methods.

**Bioinformatics analysis**		**DDA**				**DIA**			
		**SDT-B**	**SDT-U/S**	**SDT-B-U/S**	**SDT-LNG-U/S**	**SDT-B**	**SDT-U/S**	**SDT-B-U/S**	**SDT-LNG-U/S**
Molecular weight (kDa)	< 10	71 ± 2	73 ± 2	71 ± 5	71 ± 3	78 ± 0	78 ± 0	78 ± 1	77 ± 1
	10–20	327 ± 10	335 ± 2	329 ± 18	324 ± 13	327 ± 1	326 ± 1	328 ± 1	327 ± 0
	20–30	428 ± 13 ^c^	434 ± 5 ^bc^	447 ± 6 ^a^	440 ± 7 ^ab^	440 ± 1	440 ± 1	439 ± 0	439 ± 1
	30–40	407 ± 10	412 ± 9	414 ± 5	415 ± 5	399 ± 1	398 ± 1	399 ± 1	399 ± 1
	40–50	272 ± 10	275 ± 3	278 ± 1	276 ± 8	271 ± 1	271 ± 1	271 ± 1	271 ± 0
	50–60	164 ± 4	162 ± 6	165 ± 2	165 ± 1	165 ± 1	164 ± 1	165 ± 0	165 ± 1
	60–70	84 ± 2	82 ± 3	87 ± 1	85 ± 2	85 ± 1	85 ± 1	86 ± 1	85 ± 1
	70–80	60 ± 2	61 ± 1	61 ± 2	60 ± 1	63 ± 1	63 ± 1	63 ± 0	63 ± 1
	80–90	43 ± 2	42 ± 2	45 ± 1	45 ± 1	43 ± 1	43 ± 1	43 ± 0	43 ± 0
	90–100	30 ± 1	30 ± 1	31 ± 1	31 ± 1	33 ± 1	33 ± 1	33 ± 0	33 ± 1
	>100	62 ± 1	63 ± 1	62 ± 1	63 ± 1	57 ± 1	57 ± 1	57 ± 0	57 ± 0
Isoelectric point	< 4	5 ± 1	4 ± 0	4 ± 0	4 ± 1	4 ± 0	4 ± 0	4 ± 1	4 ± 0
	4–5	237 ± 3	236 ± 2	231 ± 10	235 ± 3	229 ± 0	229 ± 0	229 ± 1	228 ± 1
	5–6	876 ± 15	886 ± 11	891 ± 15	890 ± 12	887 ± 1	886 ± 0	886 ± 1	886 ± 1
	6–7	379 ± 16	378 ± 13	387 ± 3	382 ± 7	379 ± 1	378 ± 1	379 ± 1	379 ± 1
	7–8	78 ± 6	77 ± 6	83 ± 2	81 ± 1	79 ± 1	79 ± 1	79 ± 0	79 ± 0
	8–9	147 ± 6	146 ± 8	154 ± 4	153 ± 4	150 ± 0	150 ± 1	150 ± 0	149 ± 1
	9–10	173 ± 8	173 ± 8	183 ± 3	179 ± 1	180 ± 2 ^a^	180 ± 1 ^a^	147 ± 58 ^b^	179 ± 1^a^
	>10	53 ± 3	55 ± 1	57 ± 1	54 ± 2	54 ± 0	54 ± 0	54 ± 0	54 ± 0

The statistical significance of the four treatments was assessed by one-way ANOVA and indicated by letters. Groups with different letters are statistically significantly different (*P* < 0.05). Those not labeled are not significantly different.

**Figure 4:**
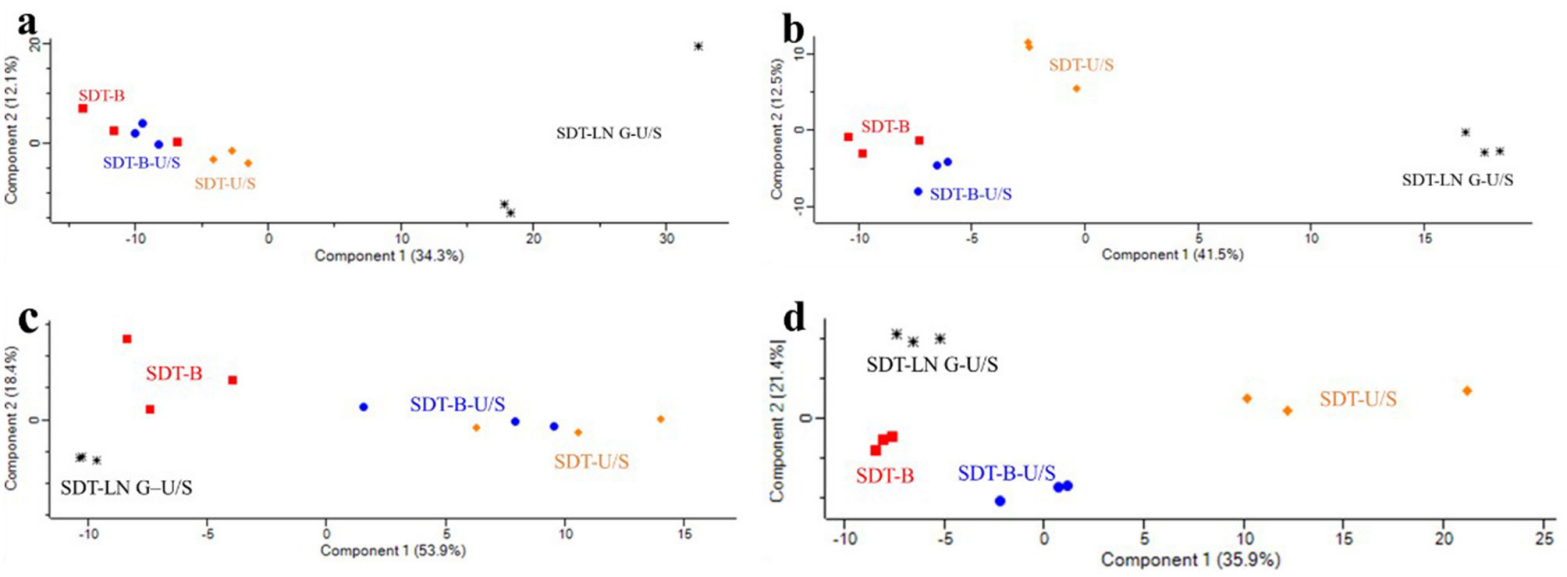
Principal component analysis of proteomic responses to different protein extraction methods. **(a)** *E. coli* identified by DDA method. **(b)** *E. coli* was identified by the DIA method. **(c)** *S. aureus* identified by DDA method. **(d)** *S. aureus* identified by the DIA method.

For the DIA analysis, a total of 1,972 proteins were identified across all four sample preparation methods, with several proteins detected in one to three methods, as depicted in the Venn diagram (Figure 4b). The MW and pI of *E. coli* proteins identified via DIA were further examined. The distribution of molecular weights among the identified proteins showed no significant variation across the four methods. However, fewer proteins with pI values ranging from 9.0 to 10.0 were identified using the SDT-B-U/S method compared to the other methods (Table 1).

### 3.3 Characterization of identified peptidess and proteins of *S. aureus*

In the DDA analysis, the SDT-B method has substantially lower number of identified peptides (8,194) compared to SDT-U/S, SDT-B-U/S, and SDT-LN G-U/S (Figure 1c). Additionally, the occurrence of peptides with 0 or 1 missed cleavage site was significantly higher form the SDT-B-U/S and SDT-LN G-U/S methods compared to the SDT-B and SDT-U/S methods (Figure 2a). However, no significant differences in the number of identified peptides and the occurrence of missed trypsin cleavage sites across the four methods based the DIA data (Figures 1d, 2d).

The Venn diagrams analysis of the identified proteins from DDA data revealed that 1,250 proteins were consistently identified across all four sample preparation methods. Comparative analysis revealed that SDT-B-U/S exhibited the highest protein coverage, missing only 30 proteins (Figure 3c). The number of proteins in the 10–40 kDa molecular weight range identified by the SDT-B and SDT-U/S methods was lower than that identified using the SDT-B-U/S and SDT-LN G-U/S methods. Regarding the pI of *S. aureus* proteins, the SDT-B method identified the fewest proteins within the 4.0–6.0 pI range compared to SDT-B-U/S and SDT-LN G-U/S methods (Table 2).

**Table 2:** The molecular weight, isoelectric point, and subcellular localization of *S. aureus* protein are identified from the four methods.

**Bioinformatics analysis**		**DDA**				**DIA**			
		**SDT-B**	**SDT-U/S**	**SDT-B-U/S**	**SDT-LNG-U/S**	**SDT-B**	**SDT-U/S**	**SDT-B-U/S**	**SDT-LNG-U/S**
Molecular weight (kDa)	< 10	81 ± 2	85 ± 1	87 ± 1	86 ± 4	74 ± 1	74 ± 1	74 ± 1	74 ± 1
	10–20	244 ± 13^c^	252 ± 11 ^bc^	271 ± 2 ^a^	262 ± 8 ^ab^	224 ± 1	225 ± 1	225 ± 0	223 ± 1
	20–30	240 ± 19 ^b^	252 ± 8 ^b^	275 ± 2 ^a^	267 ± 6 ^a^	193 ± 1	193 ± 1	194 ± 1	194 ± 1
	30–40	245 ± 16 ^c^	263 ± 8 ^b^	281 ± 2 ^a^	274 ± 2 ^ab^	200 ± 1	200 ± 1	200 ± 1	200 ± 1
	40–50	180 ± 10 ^b^	186 ± 6 ^ab^	195 ± 2 ^a^	194 ± 1 ^a^	151 ± 2	151 ± 2	151 ± 1	151 ± 2
	50–60	116 ± 5	116 ± 3	122 ± 1	121 ± 3	97 ± 2	97 ± 1	97 ± 1	97 ± 1
	60–70	56 ± 2	57 ± 3	64 ± 1	61 ± 1	45 ± 1	45 ± 1	45 ± 1	45 ± 1
	70–100	75 ± 4	79 ± 3	82 ± 2	83 ± 1	69 ± 1	69 ± 1	69 ± 1	69 ± 1
	>100	36 ± 3	37 ± 1	39 ± 1	38 ± 1	30 ± 1	30 ± 1	30 ± 1	30 ± 1
Isoelectric point	< 4	11 ± 1	10 ± 1	12 ± 1	11 ± 1	8 ± 1	8 ± 1	8 ± 1	8 ± 1
	4–5	320 ± 18 ^b^	337 ± 11 ^ab^	354 ± 2 ^a^	345 ± 7 ^a^	293 ± 1	294 ± 1	294 ± 1	293 ± 1
	5–6	487 ± 29 ^c^	505 ± 19 ^bc^	547 ± 3 ^a^	537 ± 11 ^ab^	416 ± 1	416 ± 1	417 ± 1	417 ± 1
	6–7	147 ± 29	137 ± 3	152 ± 2	146 ± 2	112 ± 1	112 ± 1	112 ± 1	111 ± 1
	7–8	39 ± 2	41 ± 1	42 ± 1	43 ± 2	31 ± 1	31 ± 1	31 ± 1	31 ± 1
	8–9	70 ± 6	76 ± 6	83 ± 2	79 ± 2	45 ± 1	45 ± 1	45 ± 1	45 ± 1
	9–10	184 ± 11	190 ± 3	197 ± 1	191 ± 6	150 ± 1	150 ± 1	150 ± 1	150 ± 1
	>10	29 ± 1	29 ± 1	30 ± 1	30 ± 1	27 ± 1	27 ± 1	27 ± 1	27 ± 1

The statistical significance of the four treatments was assessed by one-way ANOVA and indicated by letters. Groups with different letters are statistically significantly different (*P* < 0.05). Those not labeled are not significantly different.

For the DIA data, a total of 1,138 proteins were identified across all four sample preparation methods, with a small number of proteins uniquely identified by individual methods, as shown in the Venn diagram (Figure 3d). The distribution of MW and pI of the identified *S.aureus* proteins exhibited no significant variation across the four sample preparation techniques (Table 2).

### 3.4 Bioinformatics analysis of the identified proteins of *E. coli*

The PCA score plots revealed no distinct separation between the SDT-B-U/S and SDT-B samples, whereas the SDT-LN G-U/S method exhibited clear separation compared to the other methods based on the DDA dataa (Figure 4a). Additionally, PCA based on the DIA data reveals that the protein profiles of the SDT-B and SDT-B-U/S methods exhibit considerable similarity, while showing clear separation from those of the SDT-U/S and SDT-LN G-U/S methods (Figure 4b).

From the DDA data, 180 proteins showed significant differences across the four protein extraction methods. Among the four methods, the SDT-B protocol has the higher abundance of proteins such as the outer membrane protein (ompC), cold shock-like protein, and cell shape-determining protein. The SDT-U/S method exhibited elevated expression of membrane-associated proteins including the protein transport ATP-binding protein, Ni/Fe-hydrogenase 2 b-type cytochrome subunit, and O-antigen flippase. The SDT-B-U/S method was increased abundance of proteins such asompC, amino acid racemase, and ribose import permease protein. Meanwhile, the SDT-LN G-U/S method was increased abundances of proteins including fimbrial biogenesis outer membrane usher protein, anti-adapter protein, and mechanosensitive ion channel family proteins, relative to the other methods.

**Figure 5:**
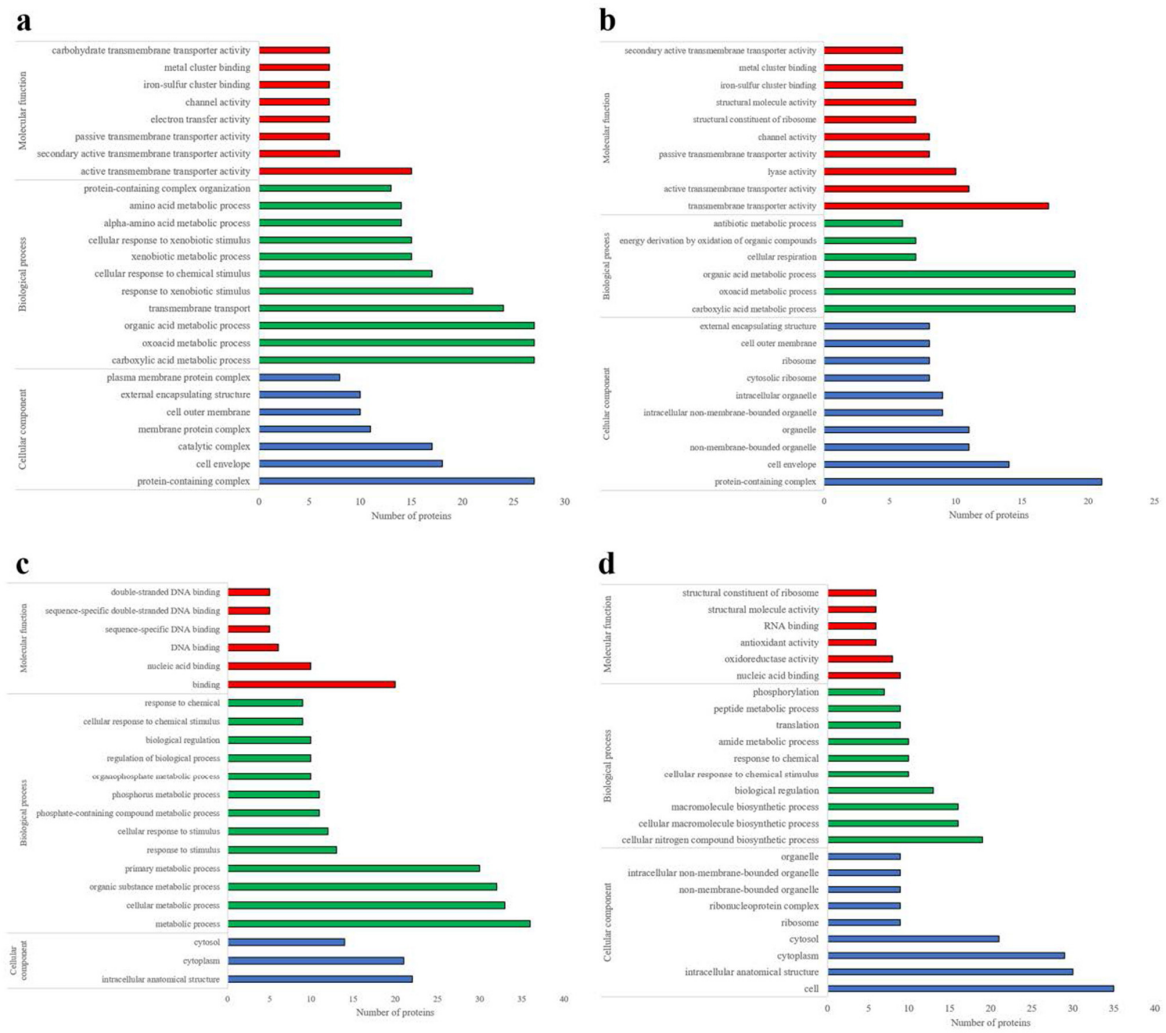
Analysis of differential protein molecular functions, biological processes, and cellular components. **(a)** *E. coli* identified by DDA method. **(b)** *E. coli* was identified by the DIA method. **(c)** *S. aureus* identified by DDA method. **(d)** *S. aureus* identified by the DIA method.

These differential abundant proteins subjected to Gene Ontology (GO) analysis were classified into three categories: cellular component, biological process, and molecular function. Regarding of cellular components classification, the differential proteins were predominantly associated with, protein-containing complexes, cell envelopes, catalytic complexes, and various membrane structures. In terms of biological processes, the primary associations were with carboxylic acid metabolism, oxyacid metabolism, organic acid metabolism, transmembrane transport, and responses to xenobiotic stimuli. Regarding of the biological functions, the proteins were mainly related to transmembrane transporter proteins and cluster binding (Figure 5).

Based on the DIA data, 133 differential abundant proteins were identified across four extraction methods. Among these, the SDT-B method exhibited significantly higher abundance of proteins such as recombination protein, bor protein, and ompC. The SDT-B-U/S method was characterized by an enrichment of proteins such as flagellin, 50S ribosomal protein (RplN), and glutamine synthetase, cellulose biosynthesis protein and ompC. Additionally, the SDT-LN G-U/S method demonstrated increased expression of proteins such as L-lactate permease, DNA-binding transcriptional activator, and sulfate adenylyl transferase subunit compared to the other extraction methods. GO analysis revealed that the differentially abundant proteins identified in the DIA data were primarily associated with GO terms that were largely consistent with those obtained from the DDA analysis (Figure 5b).

In both the DDA and DIA datasets for *E. coli*, a total of 63 differential abundant proteins were shared all methods. The membrane proteins ompX and ompC were identified at higher levels in the SDT-B and SDT-B-U/S methods compared to the other techniques. Conversely, the membrane protein ompW was found to be most highly expressed in the SDT-LN G-U/S method.

### 3.5 Bioinformatics analysis of bacterial proteins of *S. aureus*

According to the DDA data, PCA score plots revealed no distinct separation between the SDT-B-U/S and SDT-U/S samples, whereas the SDT-LN G-U/S and SDT-B samples exhibited clear separation from both SDT-B-U/S and SDT-U/S samples (Figure 4c). Similarly, PCA analysis of the DIA data demonstrated a pronounced distinction of the SDT-U/S method from the others, with the SDT-LN G-U/S, SDT-B-U/S, and SDT-B samples exhibiting relatively closer clustering (Figure 4d).

Based on the DDA data, 168 differential abundant proteins in *S. aureus*. Notably, SDT-B method exhibited a significantly higher number of proteins associated with plasma and cellular membranes compared to the other methods. 3-hydroxyacyl-[acyl carrier protein] dehydratase (FabZ), type VII secretory system protein, and glycine cleavage system H protein. The SDT-U/S and SDT-B-U/S methods were abundant proteins, including histidine-containing protein, glyoxalase/bleomycin resistance protein, and segregation and condensation protein B (rluB). Additionally, SDT-LN G-U/S demonstrated higher expression levels of proteins including tyrosine-tRNA ligase (tyrS), purine nucleoside phosphorylase, and CorA family transporter proteins.

These identified differential abundant proteins were categorized based on their. As shown in Figure 5c, the predominant molecular functions of the differential proteins, were involved in binding including nucleic acid, and deoxyribonucleic acid (DNA) binding. The most frequent biological processes were related to cellular metabolic activities, responses to stimuli, and regulatory functions. The differential abundant proteins were primarily localized in intracellular anatomical structures, particularly the cytoplasm and cytosol.

According to the DIA data, a total of 152 differential abundant proteins were identified across the extraction methods. The SDT-B method exhibited increased expression of proteins such as 3-oxoacyl-[acyl carrier protein] synthase, FabZ, and the 50S RplN. The SDT-LN G-U/S method showed higher abundance of cytoplasmic proteins, including dihydrofolate reductase and cobyric acid synthase. Additionally, the SDT-B-U/S method demonstrated elevated levels of DUF443 domain-containing protein localized to the plasma membrane.

Compared with the differential abundant proteins associated with the GO terms from the DDA data, those derived from the DIA data exhibited notable distinctions, particularly in the categories of molecular function and biological processes, as illustrated in Figure 5. Specifically, the differential abundant proteins identified by DIA were primarily associated with nucleic acid binding, oxidoreductase activity, and antioxidant activity, while the main biological processes involved in cellular nitrogen compound biosynthesis, cellular macromolecular biosynthesis, and biological regulation (Figure 5d).

A notable distinction in protein expression was observed between *S.aureus* samples subjected to sonication and those that were not. Specifically, proteins such as molybdopterin synthase sulfur carrier subunit, manganese superoxide dismutase (Fe), tyrS, and purine nucleoside phosphorylase (DeoD-type) were more prevalent in the sonicated samples. These findings suggest that sonication may have a notably impact on protein extraction in *S. aureus*.

## 4 Discussion

### 4.1 Impact of sample preparation methods on the identification of protein and peptide

In this study, the impact of four distinct protein extraction methods on the proteomes of *E. coli* and *S. aureus* using both DDA and DIA proteomic techniques were evaluated. Regarding the quantity of bacterial proteins, our findings demonstrate that the protein preparations from *E. coli* and *S. aureus* using the four methods yielded a comparable or greater number of proteins than previously reported in other studies (Fortuin et al., 2021; Xu et al., 2021). Especially, a previous study quantified over 2,000 proteins of *E. coli* across 60 diverse growth conditions—including nutrient limitations, non-metabolic stresses, and non-planktonic states—using DIA/SWATH mass spectrometry in combination with a novel protein inference algorithm (Mori et al., 2021). These investigations have significantly advanced our understanding of bacterial proteome composition by enriching insights from multiple aspects, including sample preparation strategies, mass spectrometry techniques, and bacterial protein identification. Moreover, such quantitative studies provide a valuable foundation for linking gene expression profiles to physiological states.

In the present study, the protein quantities identified from *E. coli* and *S. aureus* using the SDT-B and SDT-U/S methods did not differ significantly, which is consistent with a previous research (Livernois et al., 2009). However, in the present study, no obvious differences were observed in the proteins identified from *E. coli* and *S. aureus* using the SDT-B and SDT-U/S extraction methods. Interestingly, the SDT-B-U/S approach resulted in an increased number of identified proteins for both *E. coli* and *S. aureus*, as evidenced by the DDA data. This outcome results from the synergistic effect of boiling and ultrasonication, which function through dual mechanisms: thermal denaturation disrupts hydrophobic interactions to solubilize membrane proteins embedded within lipid bilayers, while ultrasonic cavitation generates localized shear forces that mechanically disrupt the bacterial cell wall. This combinatorial effect explains the 2.3-fold increase in transmembrane domain-containing protein identification compared to single-modality methods. Furthermore, the number of proteins identified using the SDT-LN G-U/S method was comparable to that of the SDT-B-U/S method, likely due to the improved protein extraction facilitated by liquid nitrogen grinding. Collectively, based on DDA data, the SDT-B-U/S method appears to be both cost-effective and efficient, particularly in terms of the number of proteins identified.

There were no significant differences between the DDA and DIA analyses regarding the number of peptides and proteins identified. Notably, the results from the SDT-B-U/S method were distinctly different from those produced by the other techniques, particularly when the SDT lysate underwent boiling. It was evident that the inclusion of sonication, along with other techniques, led to the identification of a greater number of proteins and peptides (Zhang et al., 2016). Thus, additional physical disruption or alternative methods significantly enhance protein extraction efficiency from bacteria, with protocols involving SDS, hot boiling, and sonication proving advantageous in microbial proteomics studies. Our analysis of peptides that did not undergo trypsin cleavage revealed that the same protein extraction method influenced the distribution of trypsin cleavage sites. These findings suggest that the reduction or alkylation of bacterial proteins, achieved through various sample preparation techniques, affects the accessibility of trypsin cleavage sites. Furthermore, this highlights the critical role both the quantity and quality of protein samples play in influencing subsequent bacterial proteomics research (Gupta et al., 2024). Ultrasonic pretreatment has been shown to improve proteolysis and enhance the efficiency of enzymatic digestion at trypsin cleavage sites (Umego et al., 2021). Notably, ultrasonic treatment led to the highest number of missed cleavage sites, with zero peptides detected in both Gram-negative and Gram-positive bacteria.

The distribution of the identified proteins by pI and MW is presented in Table 1. Consistent with previous reports, the results show that approximately 90% of the prevalent proteins in the *E. coli* proteome possess pI ranging from 4 to 7 and MW between 10 and 100 kDa (Han and Lee, 2006). Subsequently, the majority of identified proteins exhibited molecular weight predominantly within the 10 kDa to 50 kDa range, aligning with findings reported in previous studies in both *E. coli* and *S. aureus* (Yan et al., 2013). Notably, the SDT-B-U/S approach demonstrated a obvious advantage in identifying proteins within the 20 to 30 kDa range in both *E. coli* and *S. aureus* compared to the other methods. This result likely due to the pronounced effect of the boiling coupled with ultrasonic treatment, which is widely acknowledged as an effective strategy for enhancing bacterial protein extraction (Perera and Alzahrani, 2021; Luo et al., 2021).

### 4.2. Evaluation of sample preparation methods based on the comparison of DDA and DIA methods

In bottom-up proteomics, DDA plays a pivotal role in enhancing DIA by providing high-confidence peptide identifications, which serve as the foundation for spectral library construction, thereby facilitating accurate peptide extraction and quantification in DIA workflows (Guan et al., 2020). The SDT-B-U/S method, which combines heat treatment with ultrasonication, has demonstrated high efficacy in extracting membrane-associated proteins, while also minimizing missed cleavage sites, thereby making the resultant protein extracts well-suited for downstream proteomic analysis. Experimental evidence suggests that the combination of heat and ultrasound treatment is particularly beneficial for Gram-positive bacteria, such as *S. aureus*, due to their thick and rigid peptidoglycan cell walls. In this study, the SDT-B-U/S method exhibited superior extraction efficiency for *S. aureus*, whereas the use of liquid nitrogen grinding produced comparatively lower performance. In cases where spectral libraries are of critical importance, such as studies involving limited protein samples that require fractionation to maximize protein identification, DDA may offer a more advantageous and effective approach for protein identification. Based on our findings, applying heat-induced lysis coupled with ultrasonication, as implemented in the SDT-B-U/S method, is recommended, as this enhances protein extraction efficiency. Although DIA is increasingly favored for quantitative proteomics due to its reproducibility and comprehensiveness (Li et al., 2020), when it relies on high-confidence peptide identifications generated via DDA for the construction of robust and comprehensive spectral libraries (Gillet et al., 2012).

In our study, the reduced number of proteins and peptides detected via DIA compared to DDA, can be attributed to the reliance of DIA on spectral libraries derived from the DDA-generated protein database. However, PCA results showed that DIA outperforms DDA in terms of reproducibility and the accuracy of relative protein quantification, consistent with previous findings on the high reproducibility of the DIA based method (Barkovits et al., 2020). These observations highlight the necessity of developing data analysis strategies for DIA that are independent of DDA, enabling direct protein identification from DIA data. Numerous studies have already explored and applied such strategies. These methodological advancements hold the potential to expand the range of identifiable proteins, thereby contributing to a more comprehensive interpretation of the biological functions associated with the studied samples (Pino et al., 2020).

### 4.3 Evaluation of sample preparation methods based on bioinformatics comparisons

Based on GO analysis, the differential abundant proteins identified in *E.coli* and *S.aureus* were predominantly associated with transmembrane transport, transferase activity, oxidoreductase activity, and hydrolase activity. These functions are integral to key energy-generating pathways, including glycolysis and glucose metabolism, which are essential for bacterial growth and proliferation, as supported by several studies (Tian et al., 2022; Wang et al., 2018; Savijoki et al., 2020). A previous study has demonstrated that obvious changes in transmembrane transport and metabolic pathways occur in response to changes in bacterial growth conditions (Liu et al., 2020). These phyiological functions are crucial not only for energy production and membrane-associated metabolism, but also for protein transport and bacterial survival under stress, ultimately supporting cellular proliferation and adaptation to various environments (He et al., 2021a). Interestingly, sonication treatment obviously enhanced the detection of proteins related to transmembrane transport and metabolic processes. A previous study demonstrated that sonication treatment effectively disrupted the cell membranes of *E. coli*, leading to the leakage of cytoplasm proteins and and the dissociation of membrane-associated proteins (He et al., 2021b). Most proteins extracted from *E. coli* were localized in the ribosome, cytoplasm, and plasma membrane. In contrast, *S. aureus* exhibited a comparatively lower abundance of membrane-associated proteins, which may be attributed to its thicker peptidoglycan layer that potentially impedes complete cell lysis during sample preparation (Sieradzki and Tomasz, 2003). Thus, for the extraction of proteins from Gram-positive bacteria—which possess robust peptidoglycan cell walls—boiling of the lysate coupled with sonication proved effective in enhancing the recovery of extracellular and membrane-associated proteins.

During centrifugation, lysates from *S. aureus* predominantly released intracellular contents, while the cell wall and membrane fractions were precipitated, resulting in suboptimal recovery of membrane-associated proteins. This observation is consistent with the findings of Nandakumar et al. (2005), who reported that heat-based methods improved membrane proteins solubilization of *S. aureus*. In alignment with these results, our study demonstrated that the SDT-B and SDT-B-U/S methods were effective in extracting abundant membrane proteins, whereas the SDT-U/S approach was not obvious efficient.

The six most abundant proteins identified from *E. coli* and *S. aureus* using the DIA technique were subjected to detailed analysis. For *E. coli*, all four extraction strategies presented comparable results, indicating that these protocols were sufficient for high-efficiency protein recovery. Notably, the SDT-LN G-U/S method, which incorporates liquid nitrogen grinding, effectively disrupted the cell envelope of *E. coli*, facilitating the identification of membrane-associated proteins, as previously reported (Li et al., 2019). However, SDT-LN G-U/S method was suboptimal for *S. aureus*, likely due to its thick peptidoglycan cell wall, which hinders efficient cell lysis. Contrary to previous findings reported by Bai et al. (2022) and Tarrant et al. (2019), our study identified fewer membrane-associated proteins using this approach, suggesting that liquid nitrogen grinding may be less effective for membrane protein extraction in *S. aureus* under the conditions tested. Additionally, the SDT-B-U/S method effectively facilitated the extraction of proteins localized to the the extracellular region and ribosomes, as supported by our GO term analysis.

Recent studies have also reported enhanced protein identification in *S.aureus* following ultrasound-assisted extraction (Bezrukov et al., 2021; Lakshmi et al., 2022; Valliammai et al., 2020; Kirsch et al., 2020). Collectively, these findings indicate that the SDT-B-U/S method is not only straightforward to implement but also yields relatively consistent and reliable results, making it a practical choice for proteomic analysis of Gram-positive bacteria.

## 5 Conclusions

This study provides a comparative evaluation of four different bacterial protein sample preparation methods applied to *E.coli* and *S.aureus* utilizing both DDA and DIA proteomic strategies. The results indicated that the SDT-B-U/S method obviously enhanced protein and peptide identification, almost yielding the highest number of proteins and peptides in both *E. coli* and *S. aureus* under DDA analysis. Notably, the number of proteins in the 20–30 kDa range was substantially higher in samples preparation, particularly in the identification of membrane-associated proteins with the Fortuin method. The integration of orthogonal fragmentation strategies during sample preparation proved advantageous, facilitating complementary identification and quantitative characterization of the bacterial proteome. Future studies are needed to explore whether the lack of significant differences observed in the DIA data contributes to the identification outcomes observed in the DDA mode.

## Data Availability

The data presented in the study are deposited in the Mendeley Data repository, accession number https://doi.org/10.17632/p9km24dxry.1.

## Supplementary material

Supplementary Figure S1 SDS-PAGE electropherograms. **(a)** *E. coli*. **(b)** *S. aureus*. M: marker. 1, 2, 3: SDT-B. 4, 5, 6: SDT-U/S. 7, 8, 9: SDT-B-U/S. 10,11,12: SDT-LNG-U/S.
