# Correction: Evaluation of protein extraction methodologies on bacterial proteomic profiling: a comparative analysis

**DOI:** 10.3389/fmicb.2025.1673697

**Published:** 2025-09-01

**Authors:** Hongning Jiang, Aiyun Han, Yangdong Zhang, Yanxin Li, Chao Jiang, Qijing Du, Rongbo Fan, Yongxin Yang, Rongwei Han

**Affiliations:** ^1^College of Food Science and Engineering, Qingdao Agricultural University, Qingdao, China; ^2^Shijiazhuang Food Engineering Technology Innovation Center, Shijiazhuang University, Shijiazhuang, Hebei, China; ^3^Key Laboratory of Quality & Safety Control for Milk and Dairy Products of Ministry of Agriculture and Rural Affairs, Institute of Animal Sciences, Chinese Academy of Agricultural Sciences, Beijing, China; ^4^Shandong Sanyuan Dairy Co., Ltd., Weifang, Shandong, China

**Keywords:** microbial proteomics, protein extraction optimization, data-dependent acquisition, data-independent acquisition, proteomic methodology

In the published article, there was an error in **affiliation 2**. This originally read, “Shijiazhuang Food Engineering Technology Innovation Center, Shijiazhuang College, Shijiazhuang, Hebei, China”. The affiliation has been updated to read:

“Shijiazhuang Food Engineering Technology Innovation Center, Shijiazhuang University, Shijiazhuang, Hebei, China”.

In the published article, the department details were omitted from **affiliation 3**. The correct affiliation is as follows:

“Key Laboratory of Quality & Safety Control for Milk and Dairy Products of Ministry of Agriculture and Rural Affairs, Institute of Animal Sciences, Chinese Academy of Agricultural Sciences, Beijing, China”.

The original version of this article has been updated.

