# Supplementary figures and images for: Evaluation of protein extraction methodologies on bacterial proteomic profiling: a comparative analysis
Source: Front Microbiol. 2025 Jul 17;16:1586662. doi: 10.3389/fmicb.2025.1586662 (PMC12312612; doi:10.3389/fmicb.2025.1586662)

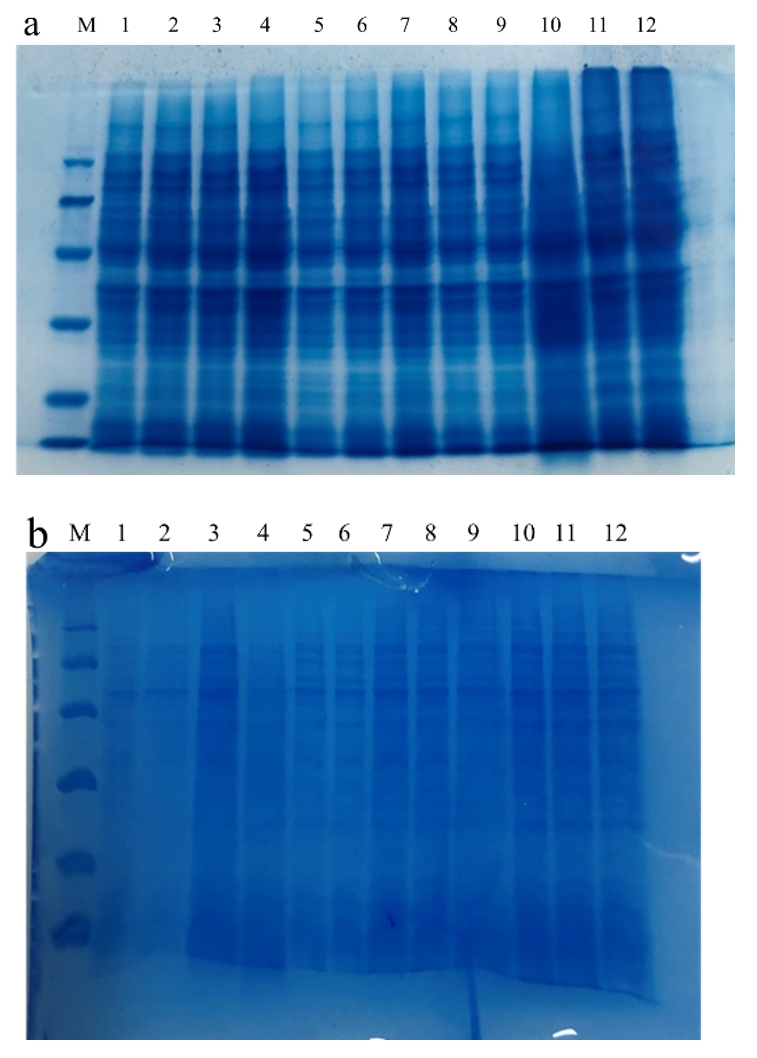

Supplement: Supplementary Figure S1 — SDS-PAGE electropherograms. (a) E. coli. (b) S. aureus. M: marker. 1, 2, 3: SDT-B. 4, 5, 6: SDT-U/S. 7, 8, 9: SDT-B-U/S. 10,11,12: SDT-LNG-U/S. [file Image_1.jpeg]
